# A Heart-Hand Syndrome Gene: *Tfap2b* Plays a Critical Role in the Development and Remodeling of Mouse Ductus Arteriosus and Limb Patterning

**DOI:** 10.1371/journal.pone.0022908

**Published:** 2011-07-29

**Authors:** Feng Zhao, Anja-Katrin Bosserhoff, Reinhard Buettner, Markus Moser

**Affiliations:** 1 Department of Pediatrics, Mount Sinai School of Medicine, New York, New York, United States of America; 2 Institute of Pathology, Molecular Pathology, University of Regensburg, Regensburg, Germany; 3 Institute of Pathology, University Hospital Cologne, Cologne, Germany; 4 Max-Planck-Institute of Biochemistry, Martinsried, Germany; Istituto Dermopatico dell'Immacolata, Italy

## Abstract

**Background:**

Patent ductus arteriosus (PDA) is one of the most common forms of congenital heart disease. Mutations in transcription factor *TFAP2B* cause Char syndrome, a human disorder characterized by PDA, facial dysmorphysm and hand anomalies. Animal research data are needed to understand the mechanisms. The aim of our study was to elucidate the pathogenesis of Char syndrome at the molecular level.

**Methodology/Principal Findings:**

Gene expression of *Tfap2b* during mouse development was studied, and newborns of Tfap2b-deficient mice were examined to identify phenotypes. Gel shift assays had been carried out to search for Tfap2 downstream genes. Promoters of candidate genes were cloned into a reporter construct and used to demonstrate their regulation by Tfap2b in cell transfection. In situ hybridizations showed that the murine transcription factor *Tfap2b* was expressed during the entire development of mouse ductus arteriosus. Histological examination of ductus arteriosus from *Tfap2b* knockout mice 6 hours after birth revealed that they were not closed. Consequently, the lungs of *Tfap2b* ^−/−^ mice demonstrated progressive congestion of the pulmonary capillaries, which was postulated to result secondarily from PDA. In addition, *Tfap2b* was expressed in the limb buds, particularly in the posterior limb field during development. Lack of *Tfap2b* resulted in bilateral postaxial accessory digits. Further study indicated that expressions of bone morphogenetic protein (*Bmp*) genes, which are reported to be involved in the limb patterning and ductal development, were altered in limb buds of Tfap2b-deficient embryos, due to direct control of *Bmp2* and *Bmp4* promoter activity by Tfap2b.

**Conclusions/Significance:**

Tfap2b plays important roles in the development of mouse ductus arteriosus and limb patterning. Loss of Tfap2b results in altered Bmp expression that may cause the heart-limb defects observed in *Tfap2b* mouse mutants and Char syndrome patients. The Tfap2b knockout mouse may add to the very limited available animal models of PDA.

## Introduction

The transcription factor TFAP2, also known as AP-2, consists of five members: TFAP2A, TFAP2B, TFAP2C, TFAP2D and TFAP2E [1], [2], [3], [4], [5], [6], [7]. TFAP2s have been found to be involved in the transcriptional regulation of many cellular genes required during embryonic development. The critical roles of TFAP2s during embryonic development have been demonstrated by phenotypes associated with natural gene mutations and knockout animal models. In *Drosophila*, several dAP-2 mutants are identified through a mutagenesis screen. Null mutants die as adults or late pupae with a reduced proboscis, severely shortened legs lacking tarsal joints, and brain abnormalities; hypomorphic alleles cause more-modest changes in leg length [8]. In mice, *Tfap2a*-deficient mice have severe anomalies, including anencephaly, body-wall defects and malformations of the outflow tract of the developing heart [9], [10], [11]. Loss of *Tfap2b* has been reported to cause congenital polycystic kidney disease due to excessive apoptosis of renal epithelial cells, ultimately resulting in terminal renal failure [12], [13]. *Tfap2c* knockout mice are arrested or retarded in their embryonic development, as they fail to establish a normal maternal-embryonic interface due to malformed extra-embryonic tissues. The majority of *Tfap2c*-null mice fail to survive beyond 8.5 days post coitum [14]. Tfap2e-null mice demonstrate disorganized olfactory bulb lamination [15]. Phenotypes associated with *TFAP2D* have not been reported.

The ductus arteriosus (DA) is an arterial connection in the fetus that shunts blood from the main pulmonary artery to the descending aorta. Ductal patency *in utero* is an active state to reduce the blood flow into the fetal lungs, which is principally maintained by prostaglandins [16]. The abrupt ductal closure at birth establishes the mature circulatory pattern. In certain animals, the DA becomes obliterated as a result of constriction and remodeling within a few hours after birth, whereas in humans it is usually complete within 48 h [17]. Failure of ductal closure, called patent ductus arteriosus (PDA), is one of the most common forms of congenital heart diseases - it affects approximately 1 in 1300 live births. If silent PDAs are included, the incidence will be at least doubled [18]. Although single gene mutations are identified in some syndromic form of PDAs [19], [20], causes for sporadic PDAs in fullterm infants remain largely unknown.

Char syndrome (CHAR; OMIM#169100) is a syndromic form of PDA with facial dysmorphism and abnormalities of the 5th finger, belonging to the class of heart-hand syndromes. It is an autosomal dominant disorder with complete penetrance, but variable expression of the phenotype. Char syndrome is linked to the chromosome 6p12–p21 and missense mutations are identified in the *TFAP2B* gene in unrelated families with genotype-phenotype correlations [21]. It is found that the mutant TFAP2B proteins act via a dominant-negative mechanism [4], [19]. In a later study, splice site mutations within the *TFAP2B* gene have been identified causing Char syndrome in a haploinsufficiency mechanism [22], [23]. The pathogenesis of this disease is unclear. In this report, we described that transcription factor Tfap2b played a critical role in the development and remodeling of mouse ductus arteriosus as well as in the limb patterning, elucidating the pathogenesis of Char syndrome on a molecular basis. Complete loss of Tfap2b caused PDA, while even partial loss could result in postaxial accessory digits. Exploration of the downstream genes demonstrated that Tfap2b regulated bone morphogenetic protein (BMP) expressions, which play very important roles in the development of both the cardiovascular system and the limbs, and may contribute to the heart-limb defects in *Tfap2b* knockout mice.

## Results

### *Tfap2b* is expressed in precursors of the DA and the aortic arch

We previously described the *Tfap2b* expression pattern during mouse development [2], [24]. Here, we focus on its unpublished expression pattern during the development of the cardiovascular system and the limbs, which are typically affected in Char syndrome patients.

**Figure 1:**
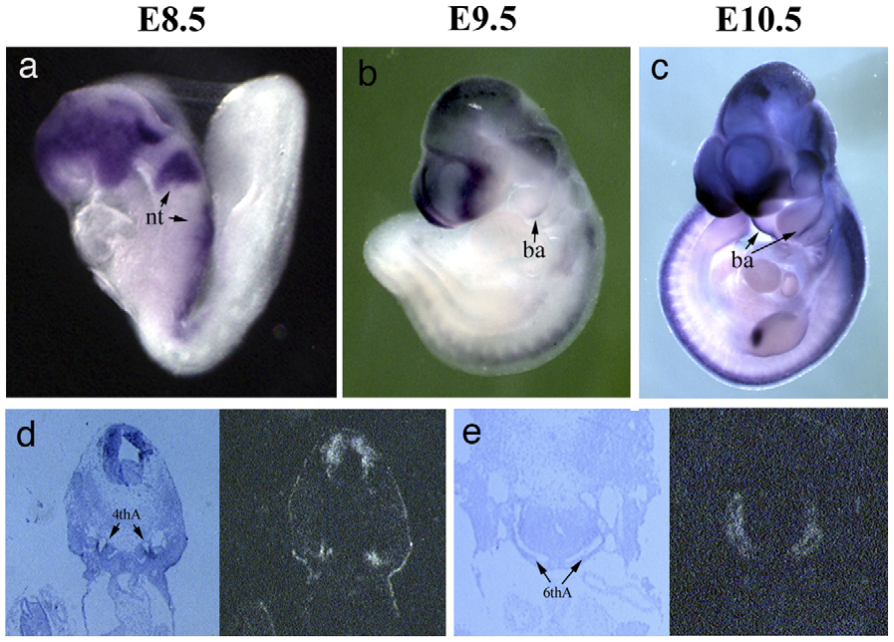
Expression of *Tfap2b* in precursors of the ductus arteriosus. Whole mount *in situ* hybridization showed *Tfap2b* expression in the neural tube as well as branchial arches at E8.5–E10.5 (a, b, c). *In situ* hybridizations with a radioactive *Tfap2b* mRNA probe on transverse sections of E11.5 embryos demonstrated *Tfap2b* expression in the 4^th^ and 6^th^ aortic arch arteries (d, e), the precursors of arch of the aorta and ductus arteriosus, respectively. The left panels were bright field photomicrographs and the right panels were dark field photomicrographs. nt, neural tube; ba, branchial arch; 4thA, the 4^th^ aortic arch artery; 6thA, the 6^th^ aortic arch artery.

The neural crest is a migratory population of cells that originates from the dorsal aspect of the neural tube and plays important roles in the embryonic development of mouse. *Tfap2b* gene expression starts at E8.5 in the neural tube, where neural crest cells originate. With migration of neural crest cells, the expression of *Tfap2b* in branchial arches was weakly detectable at E9.5 and was clearly visible at E10.5 (**Fig 1** **,** a, b, c). Identical results were also obtained from *in situ* hybridizations on sections from E9.5 and 10.5 embryos (data not shown). At this period, the branchial arch arteries (also called aortic arch arteries) develop, which partially remodel into important vascular structures such as the common carotid arteries formed by the 3rd aortic arch artery. The 4th and 6th aortic arch arteries develop into the aortic arch and the DA in the next stage, respectively. Notably, *in situ* hybridizations on transverse sections of E11.5 embryos indicated *Tfap2b* expression in the 4th and 6th aortic arch arteries (**Fig 1** **,** d, e).

### *Tfap2b* is expressed in the DA and aortic arch

**Figure 2:**
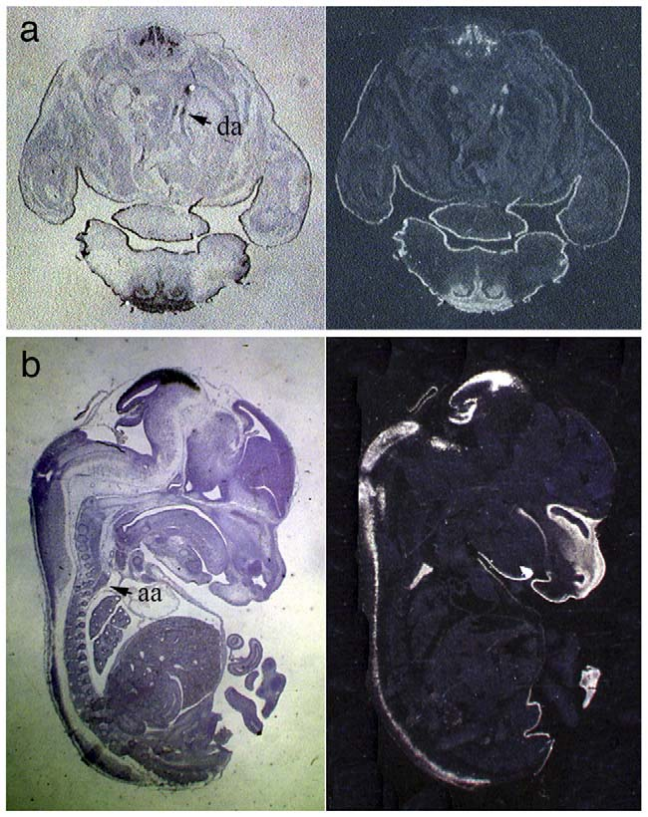
Expression of *Tfap2b* in the ductus arteriosus. *In situ* hybridization using a radioactive *Tfap2b* mRNA probe on transverse sections of E13.5 embryo showed *Tfap2b* expression in the wall of ductus arteriosus (arrow in a), while hybridization on a sagittal section of an E13.5 embryo demonstrated staining of the aortic arch (arrow in b). da, ductus arteriosus; aa, aortic arch.

Vascular system development proceeds from symmetry to asymmetry. After E12, the aortic arch and DA are formed from the left 4th and the left 6th aortic arch arteries, respectively. Fate mapping of migrating cardiac neural crest cells reveals that the wall of the ductus arteriosus derives from cardiac neural crest cells [25]. To test whether *Tfap2b* expression continues during DA and aortic arch formation, we performed *in situ* hybridizations on E13.5 embryos and detected strong *Tfap2b* expression in these structures, indicating that Tfap2b is expressed during the whole timeframe of DA and aortic arch formation (**Fig 2**). However, the expression of *Tfap2b* in the DA of late stage embryos and neonates was not able to be detected.

### Deletion of *Tfap2b* results in PDA and congestion heart failure

**Figure 3:**
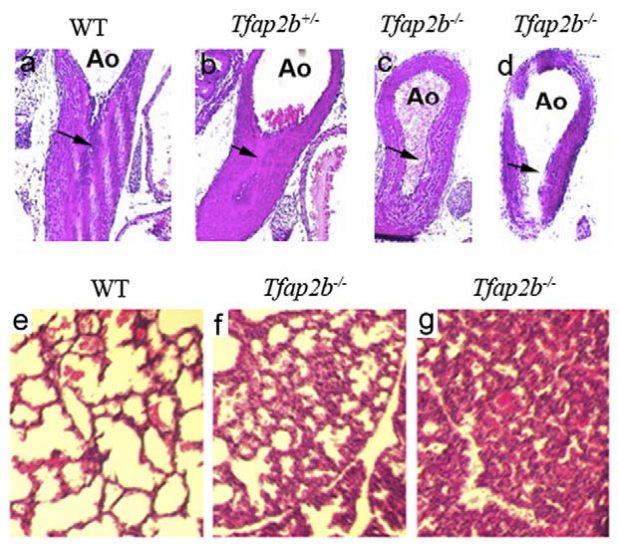
Failure of ductal closure and pathological changes in lungs of *Tfap2b* knockout mice. Representative transverse sections of DAs from WT (a), *Tfap2b* ^+/−^ (b), and two *Tfap2*b^−/−^ (c, d) pups at 6 h after birth. In each, the ductus arteriosus (DA, arrows) was inferiorly positioned and the lumen superior to the arrow was the descending aorta. The WT and *Tfap2b* ^+/−^ DAs were completely constricted, while both *Tfap2b* ^−/−^ DAs remained patent regardless of the level of section examined. ao, descending aorta. The lower panel showed the pathological changes in the lungs. The WT pup was sacrificed at 6 h after birth (e). The section in the middle was from a *Tfap2b* ^−/−^ pup that survived until 6 h and was sacrificed at 6 h (f). The third section was from a *Tfap2b* ^−/−^ neonate that died at 5 h after birth (g). Marked congestion of the pulmonary capillaries and disorganized alveolar structures were seen in the lungs of *Tfap2b* ^−/−^ mice. These pathological changes were more severe in the dead *Tfap2* ^−/−^ pups.

*Tfap2b* ^−/−^ mice developed normally through embryogenesis, but the body color of most mutants changed from pink to purple shortly after birth, showing the symptoms of heart-lung failure and lack of oxygen. Most *Tfap2b* ^−/−^ pups died within 24 h after birth, with a few of them living less than 2 h. At 2 h after birth, the majority of the *Tfap2b* ^−/−^ pups appeared to be healthy but a histological analysis of their DAs invariably showed patency (**Table 1**). In contrast, the DA of nearly all *Tfap2b* ^+/−^ and *Tfap2b^+/+^* littermates were closed at that time (p<0.01 and <0.001, respectively). Among those *Tfap2b* ^−/−^ pups that survived for at least 6 h, the DA was still not closed as shown by consecutive sections through the DA in contrast to wild-type pups (**Table 1** and **Fig 3**a, b, c, d; p<0.001).

**Table 1:** Prevalence of patent ductus arteriosus.

Genotype	PDA (2 h)	PDA (6 h)
*Tfap2b^+/+^*	0/6	0/6
*Tfap2b* ^+/−^	1/8	0/6
*Tfap2b* ^−/−^	8/8	7/7

Next, we performed histological examinations on surviving and deceased *Tfap2b* ^−/−^ mice as well as sacrificed wild-type neonate littermates. Interestingly, the lungs in *Tfap2b* ^−/−^ mice demonstrated progressive congestion of the pulmonary capillaries and a disorganized and shrunken alveolar structure, which became more severe from dying to dead pups (**Fig 3**e, f, g). As *Tfap2b* was not expressed in lungs, this phenotype was most likely secondary to the patent DA and was a sign of congestive heart failure, which might be a further cause for the early lethality of *Tfap2b* mutant mice.

### *Tfap2b* ^−/−^ mice develop postaxial accessory digits

**Figure 4:**
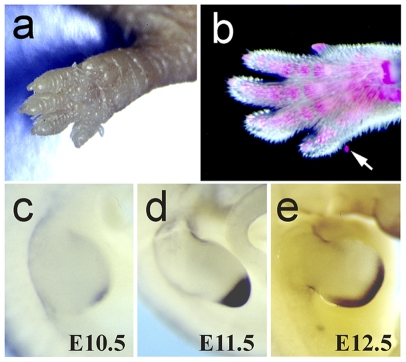
Postaxial accessory digits in *Tfap2b^−/−^* mice and *Tfap2b* expression during limb bud development. A postaxial digit-like structure grew out laterally of the fifth finger of forelimbs in all *Tfap2b* ^−/−^ mice (a). Alizarin-red staining indicated a skeletal element within the accessory digit (b). Whole mount *in situ* hybridization showed *Tfap2b* expression in the posterior region of the forelimbs at E10.5 (c), E11.5 (d) and E12.5 (e).

Tfap2b-null mice formed an additional finger-like structure that grew out laterally from the 5th finger (**Fig 4**a). This postaxial accessory digit of the forelimbs was seen in all null mutants and in 18.2% of *Tfap2* ^+/−^ mice. All wild type mice were normal (p<0.001). Hindlimbs were only affected in approximately 5% of the Tfap2b null mutants. The sizes of the accessory digit structures varied but never reached that of a normal digit. These accessory digits contained only one phalange as judged by bone staining and a finger nail never appeared (**Fig 4**b).

Abnormalities in the limbs were consistent with the gene expression patterns. *Tfap2b* expression was observed in the posterior part of the forelimb buds at E10.5 by whole mount *in situ* hybridization (**Fig 4**c). Similarly, in the hindlimbs the expression was evident in an identical pattern at E11.5. Afterwards the *Tfap2b*-expressing region in the fore- and hindlimb buds became more prominent and extended to the anterior area (**Fig 4**d, e).

### *Bmp2 and Bmp4* are Tfap2b downstream target genes that may contribute to the heart-limb defects

**Figure 5:**
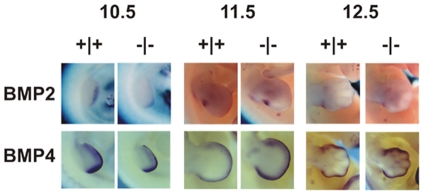
Knockout of *Tfap2b* alters the expression of *Bmp2* and *Bmp4*. Whole mount *in situ* hybridization of E10.5 to E12.5 embryos showed the expressions of *Bmp2* and *Bmp4* in the limb bud of wild type and *Tfap2b* ^−/−^ embryos. *Bmp2* expression was decreased in the limb buds of *Tfap2b* ^−/−^ embryos, particularly at E10.5, while the expression of the *Bmp4* gene was apparently increased in the limb buds of Tfap2b-null mutants.

As null mutation of *Tfap2b* caused both PDA and postaxial accessory digits in the mouse, we assumed that the absence of Tfap2b affected signaling pathways, which play important roles in the development of heart and limbs. We had explored whether Tfap2b was involved in the known prostaglandin pathway by treating pregnant mice at E18.5 with indomethacin, but observed premature closure of the DA in all wild-type and *Tfap2b* ^−/−^ fetuses, suggesting that the PDA in *Tfap2b* ^−/−^ mice was a primary event independent of the prostaglandin pathway. Bone morphogenetic proteins (BMP) have been shown to play critical roles in limb bud outgrowth and patterning [26], while BMP signaling via the type I BMP receptor, ALK2, is crucial for the development of the cardiac outflow tract and ventricular myocardium [27]. Therefore, we tested *Bmp2* and *4* expressions in the limb buds of E10.5 to E12.5 by whole mount *in situ* hybridization. Although the expression of *Bmp2* and *4* did not grossly differ in the limb buds between *Tfap2b* ^−/−^ and *Tfap2b^+/+^* embryos, we reproducibly observed a slightly broader and stronger signals of Bmp2 in *Tfap2b^+/+^* (particularly at E10.5) and Bmp4 in *Tfap2b* ^−/−^ limb buds (most prominent at E11.5) (**Fig 5**), indicating that *Bmp2* expression was downregulated and *Bmp4* expression was upregulated in the *Tfap2b*-deficient embryos comparing to that of wild type embryos.

**Figure 6:**
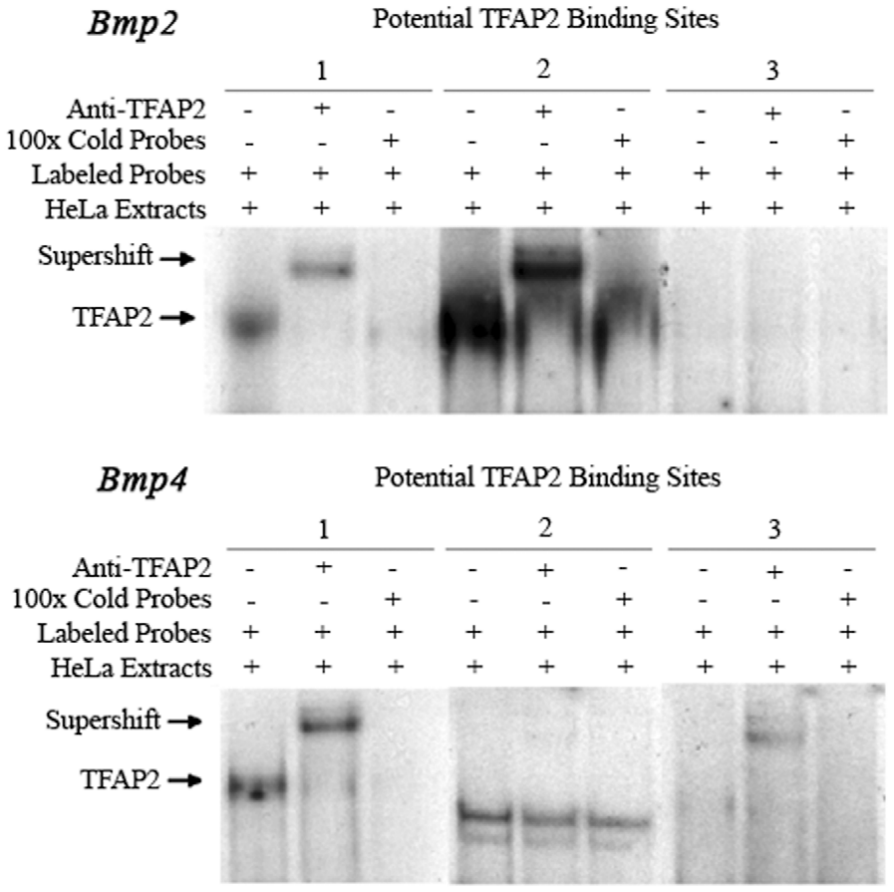
TFAP2 oligos derived from *Bmp2* and *Bmp4* promoters bind TFAP2 proteins. Electromobility gel shift assays (EMSAs) were performed by incubating HeLa cell extracts with radioactively labeled TFAP2 oligonucleotides derived from *Bmp2* and *Bmp4* gene promoters. The oligos 1, 2 in the *Bmp2* promoter and oligo 1, 3 in the *Bmp4* promoter bound TFAP2 proteins with different affinities. The signals were competed by adding 100x cold oligos and the radioactive oligos were supershifted by anti-TFAP2 antibody, demonstrating binding specificity. Oligo 2 from the *Bmp4* promoter bound unknown proteins that moved faster than the TFAP2 protein-oligo complex, but was neither competed by cold oligos nor supershifted by TFAP2 antibodies.

**Figure 7:**
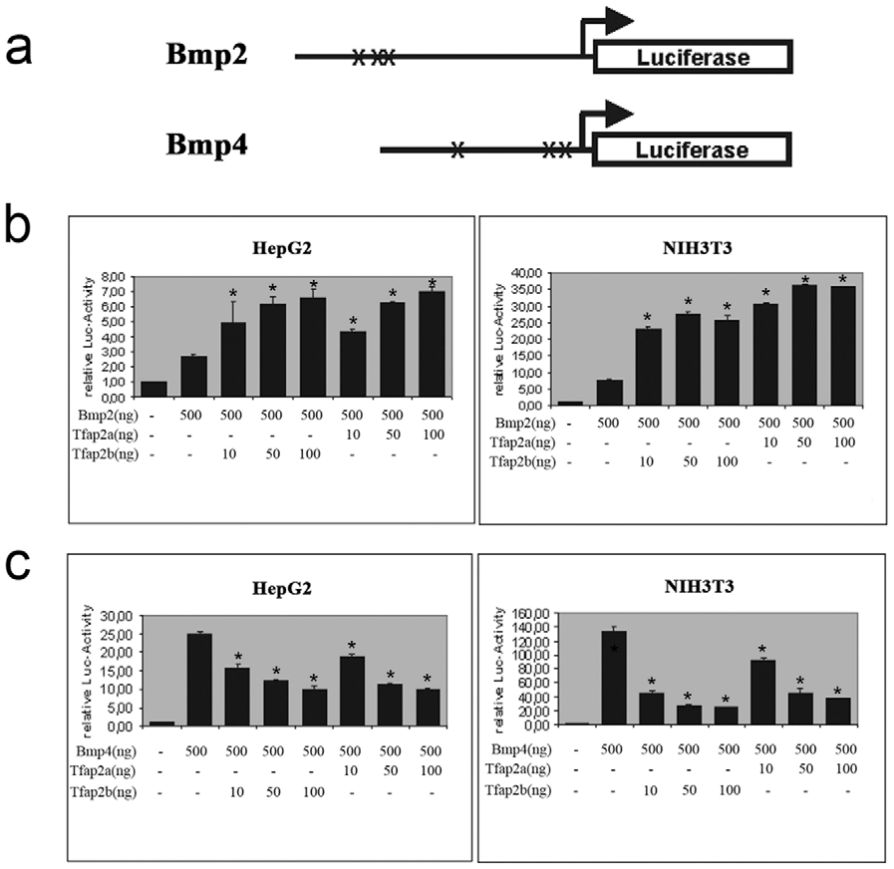
Tfap2b directly regulates *Bmp2* and *Bmp4* activities in cell transfections. The *Bmp2* and *Bmp4* promoter regions were cloned into pGL3-basic upstream of the luciferase gene as schematically shown. Crosses indicated the relative locations of the three TFAP2 binding sites in the promoters (a). Luciferase activities were increased by co-transfection of the *Bmp2* promoter construct and Tfap2 expression plasmids in HepG2 and NIH3T3 cells (b). In opposite, Luciferase activities were decreased by co-transfection of *Bmp4* promoter construct and *Tfap2* plasmids in both cell lines (c). Data in b and c were presented as mean ± SD. Stars indicated significant differences from basal levels of relative luciferase activities (P<0.01).

To test whether the *Tfap2b* gene was directly involved in the regulation of *Bmp2* and *Bmp4* gene expression, we analyzed their promoter regions. *In silico* sequence analysis revealed that both promoter regions contained at least three putative Tfap2 binding sites. These DNA-oligo sequences were synthesized, radioactively labeled and used as probes for gel shift assays. Nuclear extracts from Hela cells were used for the gel shift experiments because this cell line has been known to contain high levels of TFAP2 proteins. Among these potential TFAP2 binding sites, two oligos from the *Bmp2* promoter (first and second) and two oligos from the *Bmp4* promoter (first and third) specifically bind TFAP2 proteins, albeit with different binding affinities. Competition with cold TFAP2 oligos demonstrated that the binding was specific, while the supershift by adding anti-Tfap2 antibodies further confirmed that the probes were retarded by TFAP2 proteins (**Fig 6**). Therefore, we subcloned a 2225bp promoter fragment of the *Bmp2* gene and a 1618bp promoter fragment of the *Bmp4* gene along with their transcription start sites into the pGL-basic vector upstream of the luciferase reporter gene. Co-transfection of *Tfap2b* or *Tfap2a* expression plasmids with the promoter constructs into NIH3T3 (ATCC) or HepG4 (ATCC) cells demonstrated that *Bmp2* promoter activities were increased 3 to 6 fold in a dose dependant manner (P<0.01). In contrast, the *Bmp4* promoter activity was negatively regulated by *Tfap2* genes. Both Tfap2a and Tfap2b repressed *Bmp4* promoter activity in HepG2 and NIH3T3 cells 2.5 to 4 fold (P<0.01) (**Fig 7**).These data suggest that the transcription factor Tfap2b is involved in the regulation of Bmp2 and Bmp4 expression and changes in Bmp expression might contribute to the heart-hand defects in *Tfap2b-deficient* mice.

## Discussion

Previously we have shown that *Tfap2b* ^−/−^ mice die perinatally with massively enhanced apoptotic cell death of renal epithelial cells and terminal renal failure resulting in defective tubular secretary function and ion homeostasis [12], [13]. In this report we demonstrate that Tfap2b mutants have a patent ductus arteriosus and pathological changes in the lungs, which cause a lack of oxygen supply in *Tfap2b* ^−/−^ pups. As the kidneys, heart and lungs are all vital mouse organs, both phenotypes can lead to the death of mutant mice. Based on the physiological functions of these organs, we believe that PDA and secondary pathological lung changes should particularly account for the very early lethality of a subset of Tfap2b mutants.

Abnormalities in Char syndrome also include facial dysmorphysm and absent fifth middle phalanges with hypoplasia of the fifth proximal and distal phalanges, but no renal disease has been diagnosed [21]. Although no obvious facial dysmorphysm is observed in *Tfap2b* ^−/−^ mice, they develop PDA and postaxial hexadactyly. The finger-like structure contains a single phalange but no nail. The phenotypic difference between mouse and human might be due to different species as well as different molecular mechanisms stemming from the type of mutations. In humans two different pathological mechanisms underlying Char syndrome have been described: dominant-negative and haploinsufficiency [19], [22]. In dominant-negative mutations, the mutant proteins dimerize with and knockdown the normal TFAP2B proteins, leaving only ¼ of the functional TFAP2B level. Haploinsufficiency is caused by abnormal splicing of the *TFAP2B* gene, producing exon skipping and frameshift mutations which are expected to create a premature stop codon and bring about a nonsense-mediated decay of the transcripts that result in loss of half of the functional TFAP2B proteins. The Tfap2b-deficient mouse belongs to the haploinsufficienncy model. With a small fraction of *Tfap2b* ^+/−^ mice developing limb anomalies and PDA, a gene dose effect is observed. Since *Tfap2b* ^+/−^ mice do not develop any kidney defect, and even with ¼ of functional TFAP2B proteins in Char syndrome patients no renal abnormality is diagnosed, we postulate that the ductus arteriosus and the posterior limb field are more sensitive to reduced Tfap2b protein levels than the kidney.

Several pieces of evidence have addressed the importance of Tfap2 transcription factors in the development of the cardiovascular system. *Tfap2a* ^−/−^ mice show malformations of the outflow tract of the developing heart, with majority of them having double outlet right ventricle and a small fraction demonstrating persistent truncus arteriosus [11]. Loss of CITED2, a Tfap2 co-activator in mice, causes similar cardiac defects, confirming that transcriptional gene regulation by Tfap2 proteins is crucial for normal cardiac development [28]. In this study we demonstrate that Tfap2b is expressed in cardiac neural crest cells during the early development of mouse DA and may therefore play an essential role for its proper formation and remodeling. Both Tfap2a and Tfap2b are expressed in cardiac neural crest cells in the pharyngeal arches surrounding the pharyngeal arch arteries [11], which later populate the aortopulmonary septum and conotruncal cushions prior to and during overt septation of the outflow tract [25]. As Tfap2a and Tfap2b knockout mice show different cardiac phenotypes, it is most likely that the two genes play different roles within cardiac neural crest cells.

Tfap2b is expressed in the DA precursor and the wall of DA of middle stage mouse embryos, implying that it plays an important role as a transcription factor by regulating the expression of other genes during the early stages of ductal development, which are essential for ductal remodeling and closure at birth. By treating mutant Tfap2b fetuses with indomethacin we can exclude the connection of Tfap2b to the prostaglandin pathway. Ivey *et al* recently also have shown that Tfap2b is expressed in the vascular smooth muscle layer of the ductus arteriosus, which derive largely from neural crest cells and initiate postnatal ductal closure. Their data suggest that a transcriptional network composed of Tfap2b, Ets-1 and Hif2α may regulate ductal smooth muscle development and that a disruption of this pathway may contribute to patent ductus arteriosus by affecting the development of its smooth muscle layer [29].

Hand anomalies are another prominent characteristic of Char syndrome, causing it to be a member of the class of combined congenital cardiac and limb deformities – the so-called heart-hand syndromes. Tfap2b is restrictively expressed in the posterior mesenchyme of the developing mouse limb at E10.5 and its expression extends more anterior at later developmental stages. Consistently, hand anomalies affect only posterior digits in Char syndrome patients and *Tfap2b* ^−/−^ mice. In contrast, Tfap2a is expressed in the anterior and posterior mesenchyme. Inactivation of Tfap2a in mice result in severe limb anomalies with poly- or polysyndactylies, as well as an absence or duplication of more distal limb elements [10], [30]. These data imply that both transcription factors fulfill critical and non-redundant functions during limb bud development as they cannot compensate for the loss of the other in the posterior mesenchyme.

The mechanisms underlying the association of heart-hand defects are unknown. More than 100 Mendelian disorders present with both heart and limb defects. A hypothesis has been proposed that there is a cardiomelic developmental field – the heart and limb primordia, in the early embryo. Abnormalities in this field might result in heart-hand defects [31]. As we have documented in this study that *Tfap2b* is expressed during the entire developmental time window of the DA and the limbs, the developmental abnormalities in the ductus and limbs should be attributed to the altered expression of downstream genes, and may be through the same pathway. Bone morphogenetic proteins (BMPs) are secreted, multi-functional growth factors that belong to the transforming growth factor β (TGFβ) superfamily. Members of BMPs, particularly the BMP2 and BMP4, are expressed in the vertebrate limb bud and BMP signaling plays an important role in limb outgrowth and patterning. *Bmp* mutant mice show malformed limbs, including postaxial polydactyly with digit five duplications [26], [32], [33]. BMP2 and BMP4 have also been implicated in promoting neural crest cells' (NCC) induction, maintenance, migration and differentiation in several different model organisms [34]. The mouse has three known BMP2/4 type I receptors, of which *Bmpr1a* is expressed in the neural tube sufficiently early to be involved in neural crest development from the outset. Ablation of *Bmpr1a* caused a shortened cardiac outflow tract with defective septation, acute heart failure, and reduced proliferation of ventricular myocardium [27]. Importantly, abnormal regression of the left 6th aortic arch artery, which is the precursor of DA, was identified in mice lacking the Bmp receptor ALK2 in neural crest cells [35]. These observations provide direct evidence that Bmp signaling is required for the development of mouse DA and limbs. As the direct regulator of *Bmp2* and *Bmp4*, Tfap2b protein level and activity will alter the activities of Bmp2 and Bmp4, which will subsequently impact the development of mouse DA and limbs. Based on the literature and our findings, we conclude that *Bmp2* and *Bmp4* are Tfap2b downstream target genes and may contribute to the PDA and postaxial accessory digits in the Tfap2b-null mice.

## Materials and Methods

### Tfap2b-null mice and genotype analysis

The *Tfap2b*-deficient mice were created by disrupting the 4th exon of the *Tfap2b* gene. Tails of adult mice were cut at three weeks after birth. Tissues from newborn pups were collected at the time of sacrifice. Genomic DNA was extracted from these tissues using a DNeasy Blood & Tissue Kit (Qiagen). The genotypes for *Tfap2b* were determined by PCR using the following primers: MAP2B4d: 5′ CCT CCC AAA TCT GTG ACT TCT 3′; MAP2B4r: 5′ TTC TGA GGA CGC CGC CCA GG 3′; PGKpolyA-d: 5′ CTG CTC TTT ACT GAA GGC TCT TT 3′ [12], [13]. Control *Tfap2b^+/+^* mice were generated by crossing *Tfap2b* ^+/−^ mice to ensure an identical genetic background.

This study conforms to the Guidelines for Care and Use of Laboratory Animals published by the US National Institutes of Health. All experimental manipulations of mice were approved by the Institutional Animal Care and Use Committee of Mount Sinai School of Medicine (Approval number 98–777).

### *In situ* hybridization

A 326-bp fragment of *Tfap2b* (nt343–668) cloned in a pBS vector was used to generate a mRNA probe used for studying the *Tfap2b* expression patterns during mouse development. 20 µg plasmid DNA were linearized with suitable restriction sites to allow the T3 or T7 promoter to transcribe α35S-UTP- labeled antisense and sense *Tfap2b*-specific RNA probes. Probe length was reduced by alkaline hydrolysis at 60°C for 35 minutes. The probe was purified with a Sephadex-G50 column. For whole mount *in situ* hybridization, the RNA probes were labeled with digoxin and precipitated with ethanol. After washing with 70% ethanol, the probes were resuspended in RNase-free water.

Wild type mice, which were generated by crossing the Tfap2b^+/−^ mice, were mated overnight. The next morning, females were checked for vaginal plugs. Positive females were separated to new cages and the afternoon of the plug date was assigned a gestational 0.5 days post-coitum (dpc). Embryos were collected between 7.5 dpc and 18.5 dpc. Pregnant females were sacrificed by CO2 and the embryos were collected in DEPC-treated PBS. Embryos were fixed in 4% paraformaldehyde overnight, washed in 150 mM NaCL, and dehydrated through graded ethanol. After treating with Americlear (Allegiance) twice, embryos were embedded in Paraplast (Fisher) overnight under vacuum. Serial sections of 7 µm were cut and floated onto Superfrost/Plus (Fisher) slides. Then the slides were dried at 37°C overnight and stored at room temperature until use.

Paraffin sections of mouse embryos were deparaffinized with Americlear (Allegiance) and hydrated with graded ethanol from 100% to 95%, 85%, 70%, 50% and 30% for 3 min each step. PBS was the last step in hydration. After fixing with 4% paraformaldehyde, protease K digestion was performed, followed by treatment with TEA/acetic anhydride. Then the sections were dehydrated through a graded ethanol series (the order of graded ethanol was reversed). Hybridization was carried out at 50°C. Following washing, the sections were air dried. Autoradiography was performed by dipping the slides in the Kodak NBT2 emulsion, air drying and exposing for 7–10 days. This was followed by developing in Kodak D19 and H&E counter staining.

For whole mount *in situ* hybridization, paraformaldehyde-fixed embryos were washed with PBS/Tween-20 and then digested with protease K. After post-fixing with 4% paraformaldehyde/0.1% glutaraldehyde, hybridization was performed using a DIG-labeled RNA probe for 48 h, followed by reaction with an AP-anti-DIG antibody (Roche). Embryos were washed extensively and then incubated with BMP-purple substrate (Roche).

### Phenotype analysis

*Tfap2b* ^+/−^ males and females were mated and pregnant females were monitored until natural delivery at term. Two or six hours after birth, newborn pups from heterozygous intercrosses were decapitated and transected below the rib cages. The chests were fixed with 2% formalin for 48 h, processed with an automatic processor and paraffin-embedded. Serial sectioning was performed and the slides were stained with H&E for pathological analysis of DA. For the histological analysis of limbs, tissues were fixed in 4% paraformaldehyde/PBS over night at 4°C, then dehydrated and embedded in paraffin. Five to seven-micron sections were made with a microtome and stained with H&E.

### Promoter constructs, transfections and luciferase assays

Lambda phage and Pac libraries were screened. The 5′ end flanking regions of the murine *Bmp2* and *Bmp4* genes from residue −834 to +1394 of *Bmp2* and −1492 to +120 of *Bmp4,* referring to the transcription start site, were amplified by either genomic PCR or lambda phage screening. All fragments were inserted into the plasmid pGL3-basic (Promega) and confirmed by sequencing.

HepG2 (ATCC, HB-8065) and NIH3T3 (ATCC, CCL-92) cells were grown at 37°C / 5% CO2 in DMEM (Dulbecco's modified Eagle medium; Gibco) supplemented with penicillin (100 U/ml), streptomycin (10 µg/ml) (Sigma) and 10% fetal calf serum (Gibco). 2×105 HepG2 or 6×104 NIH3T3 cells were seeded into each well of a 6-well plate and transiently transfected with 0.5 µg promoter plasmid DNA using the Lipofectamine Plus method (Gibco) according to the manufacturer's instructions. In addition, different amounts (10, 50, 100 ng) of *Tfap2a* and *Tfap2b* cDNAs were cotransfected. All transfections were performed in triplicate. 24 hours after transfection, the cells were lysed and the luciferase activity in the lysate was measured. To normalize transfection efficiency, 0.1 µg of a pRL-TK plasmid (Promega) was cotransfected and renilla luciferase activity was measured by a Dual Luciferase Reporter Assay (Promega). *Student t* test was carried out to analyze the differences of relative luciferase activities between co-transfections with *Tfap2* plasmids and their corresponding basal levels (*Bmp* promoter construct alone).

### Gel mobility shift assays

The following complementary oligonucleotides corresponding to three AP-2 consensus binding sites in the *Bmp2* and *Bmp4* promoter regions were synthesized, annealed, radioactively end labeled with 32P and used as probes: Bmp2 AP-2 I sense: GAG TGA GCG CCC AAG GCG AGC GGG C, antisense: GCC CGC TCG CCT TGG GCG CTC ACT C; Bmp2 AP-2 II sense: GAC ACT TGG CCC GAG GGC TCG GAG C, antisense: GCT CCG AGC CCT CGG GCC AAG TGT C; Bmp2 AP-2 III sense: GCG CCG CAG CCG TGC GGG CTC TGC TG, antisense: CAG CAG AGC CCG CAC GGC TGC GGC GC; Bmp4 AP-2 I sense: AAA AAG GGG CCA AAG GGC ACT TTG T, antisense: ACA AAG TGC CCT TTG GCC CCT TTT T; Bmp4 AP-2 II sense: GAG GCG AGG CCC CGT GGC TGG ATG GG, antisense: CCC ATC CAG CCA CGG GGC CTC GCC TC; Bmp4 AP-2 III sense: AGG GAG GGG CCG CTG GGG GGA AAG A, antisense: TCT TTC CCC CCA GCG GCC CCT CCC T. Nuclear extracts were prepared from Hela cells, which are rich in TFAP2A. 20 µl reactions were set up on ice containing 6.5 µl H_2_O, 1 µl Hela cell extracts, 1 µl dI/dC (10 units/ml), 11.5 µl 2x gel shift buffer (20 mM Tris pH7.9, 9% Ficoll 400, 120 mM KCl, 8 mM MgCl2, 0.2 Mm EDTA, 100 µg/ml BSA, 0.4% NP40 and 2 mM DTT). After 15 min, the reactions were fractionated on 4% acrylamide gel. The gel was dried and visualized by autoradiography. To test the specificity, 100-fold excess of unlabeled, well-characterized TFAP2 binding oligos (AGT AGA AGC TGG GCC CCA GGC GTG GCG CTT) were added for the competition assay. Specific Tfap2-DNA complexes were determined by supershift experiments, which were performed using an anti-AP-2α antibody (Geneka).
